# Laborious but Elaborate: The Benefits of Really Studying Team Dynamics

**DOI:** 10.3389/fpsyg.2019.01478

**Published:** 2019-06-28

**Authors:** Michaela Kolbe, Margarete Boos

**Affiliations:** ^1^Simulation Center, University Hospital Zurich, Zurich, Switzerland; ^2^Institute for Psychology, University of Göttingen, Göttingen, Germany

**Keywords:** team process, team dynamics, interaction analysis, methods, measurement

## Abstract

In this manuscript we discuss the consequences of methodological choices when studying team processes “in the wild.” We chose teams in healthcare as the application because teamwork cannot only save lives but the processes constituting effective teamwork in healthcare are prototypical for teamwork as they range from decision-making (e.g., in multidisciplinary decision-making boards in cancer care) to leadership and coordination (e.g., in fast-paced, acute-care settings in trauma, surgery and anesthesia) to reflection and learning (e.g., in post-event clinical debriefings). We draw upon recently emphasized critique that much empirical team research has focused on describing team states rather than investigating how team processes dynamically unfurl over time and how these dynamics predict team outcomes. This focus on statics instead of dynamics limits the gain of applicable knowledge on team functioning in organizations. We first describe three examples from healthcare that reflect the importance, scope, and challenges of teamwork: multidisciplinary decision-making boards, fast-paced, acute care settings, and post-event clinical team debriefings. Second, we put the methodological approaches of how teamwork in these representative examples has mostly been studied centerstage (i.e., using mainly surveys, database reviews, and rating tools) and highlight how the resulting findings provide only limited insights into the actual team processes and the quality thereof, leaving little room for identifying and targeting success factors. Third, we discuss how methodical approaches that take dynamics into account (i.e., event- and time-based behavior observation and micro-level coding, social sensor-based measurement) would contribute to the science of teams by providing actionable knowledge about interaction processes of successful teamwork.

## Introduction

Modern organizations rely on teams (Edmondson, 2012; Salas et al., 2013b; Mathieu et al., 2014). For decades, team researchers have been studying how teams create and maintain high performance, how they learn, and how they satisfy their members’ needs. A remarkable finding of this research is that high team performance is not so much predicted by how able single team members are but by the way they cooperate with one another: the team process (West, 2004; Woolley et al., 2010, 2015). Team process is defined as “members’ interdependent acts that convert inputs to outcomes through cognitive, verbal, and behavioral activities directed toward organizing taskwork to achieve collective goals” (Marks et al., 2001, p. 357). This definition implies that team processes are actually dynamic, emerging over time, and changing their pattern. It stands in contrast to the way teams have mostly been studied: much empirical team research has been static rather than dynamic, assessing team states rather than exploring how team processes dynamically develop over time and how these dynamics are related to team outcomes such as performance, satisfaction, and learning (Roe, 2008; Cronin et al., 2011; Humphrey and Aime, 2014; Mathieu et al., 2014; Kozlowski, 2015). As such, much team research has relied on self-reported and cross-sectional data with small samples and short analysis periods rather than on more meaningful, time-based behavioral data. While the number of theories and concepts factoring in time and temporal dynamics in team research is rising (McGrath and Tschan, 2004; Ballard et al., 2008; Lehmann-Willenbrock, 2017), the number of published empirical studies actually integrating dynamics is small considering for how long and how urgently this research has been requested (Stachowski et al., 2009; Tschan et al., 2009, 2015; Grote et al., 2010; Lehmann-Willenbrock et al., 2011, 2013; Zijlstra et al., 2012; Boos et al., 2014; Kolbe et al., 2014; Lei et al., 2016). This may be due to both the “unease of the psychologist in face of interaction” (Graumann, 1979) as well as to methodological challenges. However, recent team research has revealed that team members’ interaction patterns rather than the frequencies of their individual actions are what discriminates higher- from lower-performing teams (Kim et al., 2012; Zijlstra et al., 2012; Kolbe et al., 2014; Lei et al., 2016). These distinguishing dynamics cannot be uncovered with static research but require process-related methods like sequential analysis, time series analysis or process modeling. It is critical to understand how team processes emerge and change and what they need and do to achieve best outcomes. This is specifically important in light of the evidence showing that poor teamwork in high-risk/high complexity fields such as healthcare can have disastrous consequences, i.e., loss of a patient’s life (Cooper et al., 1984; Flin and Mitchell, 2009; Reynard et al., 2009; Fernandez Castelao et al., 2011; Salas and Frush, 2013; Salas et al., 2013b).

In this manuscript, we use teams in healthcare as the application context for illustrating the consequences of methodological choices in studying teams. We deliberately chose healthcare as application context for three reasons. First, teamwork can save lives (Rosen et al., 2018a). There is vast evidence demonstrating that poor teamwork has been involved in medical error (Gawande et al., 2003; Greenberg et al., 2007). Improving teamwork is a major initiative in patient safety and healthcare (Pronovost, 2013; Salas and Frush, 2013; Vincent and Amalberti, 2016). Second, the processes constituting effective teamwork in healthcare are prototypical for teamwork in general: they range from decision-making (e.g., in multidisciplinary decision-making boards in cancer care) to leadership and coordination (e.g., in fast-paced, acute-care settings in trauma, surgery and anesthesia) to reflection and learning (e.g., in post-event, clinical debriefings). Many of the research gaps and much of the knowledge gained from studying teams in healthcare is applicable to teams in other industries (Salas et al., 2013b). Third, given the broad occurrence and critical importance of teams not only in healthcare, knowledge must be gained on what contributes to effective teamwork. Team science has not only a lot to offer with respect to theory and methodology, it has also an obligation to contribute to improving teamwork by providing theoretical and methodological knowledge and supporting teams in healthcare.

The goal of this manuscript is to illustrate the consequences of methodological choices when attempting to study and measure team processes “in the wild” such as in healthcare (Rosen et al., 2012; Salas, 2016). In particular, we aim to show that using methods relying on summative, cross-sectional data collection (e.g., rating teamwork aspects after a medical team performance episode) will result in limited insights into the actual dynamic team process. Instead, gaining critical comprehension of dynamics that characterize effective teamwork requires methods that are more laborious (e.g., real-time behavior coding during the medical team performance episode) but provide more elaborate understanding of what happened while working together. We argue that static team research is a methodical choice that diminishes rather than enhances potential contributions to the science of teams. While we greatly appreciate the value of teamwork surveys such as the Team Diagnostic Survey (Wageman et al., 2005) and the Aston Team Performance Inventory (West et al., 2006), particularly for assessing team members’ subjective perspective of team process functioning for the purpose of training and reflection, we argue that studying team dynamics by means of dynamic teamwork measures is a better methodological fit (Edmondson and McManus, 2007) and more promising for teamwork interventions.

For this purpose, we first describe three examples representing typical teamwork in healthcare and briefly refer to both team conceptual foundation underlying these examples and current research needs, also in order to highlight their representativeness for teamwork in general. Second, we put the methodological approaches of how teamwork in these representative examples has mostly been studied centerstage and highlight the respective consequences. Third, we illustrate potential other methodological approaches which are, for the time being, more extensive but provide benefits for applied team science.

## Three Representative Examples for Teamwork

As prototypical examples for teamwork we chose three team settings from healthcare: (1) multidisciplinary decision-making boards, (2) fast-paced, acute care settings, and (3) post-event, clinical team debriefings. The examples convey the criticality of both *teamwork* for a range of tasks in an important professional sector as well as of team *process* as a mediator between input and outcome of teamwork. All three examples represent contemporary forms of more or less *ad hoc* team constellations (Tannenbaum et al., 2012). Embedded in organizational structures, they highlight the dynamic and emergent features of teams and the resulting requirements for appropriate methods in order to grasp these features.

### Example 1: Multidisciplinary Decision-Making Boards

Multidisciplinary decision-making boards are a prototype of diverse teams in complex organizations for which the successful exchange of expertise should result in synergy. The most common example is the multidisciplinary tumor board in cancer care (Homayounfar et al., 2015) where experts of multiple disciplines discuss individual patient cases. More recently, Heart Teams have been formed consisting of experts from disciplines involved in management of complex, severe heart diseases (e.g., cardiologists, cardiac surgeons, imaging specialists, anesthesiologists and, if required, general practitioners, geriatricians, and intensive care specialists) and should find optimal treatments (Seiffert et al., 2013; Antonides et al., 2017; Falk et al., 2017). Multidisciplinary decision-making boards are implemented as countermeasure to the increasing complexity of treatment options. Their objective is to provide patients with the most effective treatment in light of the severity of the disease, patients’ requests, resources, and the current state of medical research. Multidisciplinary tumor boards have already become an international standard of cancer care (Pox et al., 2013). Heart Teams are recommended by the European Society of Cardiology and the European Association of Cardio-Thoracic Surgery (Falk et al., 2017).

The criticality of team process in multidisciplinary decision-making boards is illustrated in a meeting situation in Table 1. It shows that a lack of evidence-based communication rules, professional facilitation, and participative leadership behavior that take into account task complexity, conflicting goals, hierarchical structure, and time pressure can jeopardize the effective functioning, synergy, and development of multidisciplinary decision-making boards, and thus their ultimate mission to enhance patient care (Kolbe et al., 2019). As a consequence, team science must provide insights into effective teamwork processes as well as respective countermeasures.

**TABLE 1:** Example of a problematic teamwork situation in multidisciplinary decision-making boards.

**Situation**	**Potential teamwork process problems**	**Required teamwork process insights**
During a tumor board meeting, the chief of surgery arrives late while the discussion of a particular patient initially referred to her department has already started with a preliminary vote for inclusion into a new clinical trial instead of surgery. Using her dominant character she states that the patient will have to get surgery. None of the other board participants repeated the previously discussed arguments favoring the clinical trial and in the protocol a vote for surgery was documented as concordant decision.	Counterproductive meeting behaviors and lack of meeting rules (Allen et al., 2015).	Identification of actions required to set up and facilitate multidisciplinary tumor board meetings.
	Risk that leaders dominate discussion (Larson et al., 1998).	Understanding of facilitation techniques which allow for balanced exploitation of information from all board members and of optimal decision rules.
	Lack of psychological safety and lack of sharing information, opinions, and concerns by all board members (Mesmer-Magnus and DeChurch, 2009; Edmondson and Lei, 2014).	Understanding how to establish and maintain psychological safety during interdisciplinary tumor board meetings.

### Example 2: Fast-Paced, Acute Care Settings

Fast-paced, acute care settings such as medical emergencies are prototypical for so-called action teams, i.e., teams that are confronted with highly dynamic, complex, and consequential tasks (Tschan et al., 2006, 2011a). They require teamwork at its best (Driskell et al., 2018; Maynard et al., 2018). For example, resuscitating a patient requires prompt and well-coordinated actions such as diagnosing the cardiac arrest, oxygenating the brain and reestablishing spontaneous circulation (Tschan et al., 2011b). Other fast-paced, acute care settings require more sense-making processes, for example when the diagnosis is not yet clear. Team members must adaptively engage in immediate problem awareness and diagnosis, information-processing, problem-solving, and coordination of actions (Hunziker et al., 2011; Tschan et al., 2011a, 2011b, 2014). They must do this under time pressure and high workload—and in many instances off the cuff as *ad hoc* action teams (Kolbe et al., 2013a). Both the European Resuscitation Council (ERC) and the American Heart Association (AHA) recommend integrating teamwork trainings into advanced life support education (Bhanji et al., 2010; Soar et al., 2010). This is, in part, realized by simulation-based team training (Kolbe et al., 2013b; Salas et al., 2013a; Weaver et al., 2014).

The criticality of team process in fast-paced, acute care settings is illustrated in a sample situation in Table 2. This example highlights teamwork problems that are particularly challenging if teams face complex tasks, unpredictable circumstances, time pressure, high risk and/or rapid workload changes as it is the case in action teams.

**TABLE 2:** Example of a problematic teamwork situation in fast-paced, acute care settings.

**Situation**	**Potential teamwork process problems**	**Required teamwork process insights**
At 2 a.m. a patient is being brought into the trauma center. She appears to have multiple traumatic injuries. The nurses prepare the patient as quickly as possible and the anesthesia sub-team begins with inducting of anesthesia. The trauma doors open, the attending trauma surgeon comes in and starts yelling and forcefully expressing her disapproval that the patient lies uncovered, bare, and fully exposed in the cold room and that she wouldn’t know how many more times she has to complain about it until the nurses would eventually get it. The nurses look at each other, roll their eyes, and continue their work. So does the anesthesiologist.	High frequency of uncivil behavior and its detrimental and contagiously spreading effects for team performance outcomes (Porath and Erez, 2009; Riskin et al., 2015; Foulk et al., 2016; Bar-David, 2018; Klingberg et al., 2018).	Insights into the unfolding of incivility during fast-paced, acute care settings and into potential triggers of civility.
	Low frequency of voice behavior and related missed opportunities for improvement (Morrison and Milliken, 2000; Kobayashi et al., 2006; Detert and Burris, 2007; Tangirala and Ramanujam, 2012; Schwappach and Gehring, 2014; Raemer et al., 2016).	Understanding of social dynamics enabling voice behavior during fast-paced, acute care settings.
	Difficulty to function as highly interdependent team because of low civility (Salas, 2016).	Identification of team adaptation mechanisms for maintaining and regaining functionality despite low civility.

### Example 3: Clinical Team Debriefings

Designed to promote learning from reflected experience, debriefings are guided conversations that facilitate the understanding of the relationship among events, actions, thought and feeling processes, and team performance outcomes (Ellis and Davidi, 2005; Rudolph et al., 2007). With respect to the team setting, debriefings have some characteristics in common with the multidisciplinary decision-making boards (example 1): they rely on psychological safety for providing a conversational climate which allows for information-sharing and sense-making. They are also formed *ad hoc*, consist of interprofessional, and in many cases, multidisciplinary members across the authority gradient and exist within complex, hierarchical organizations. What distinguishes them from multidisciplinary decision-making boards is their task: whereas the boards’ task is to make decisions regarding future diagnosis and treatment, the task of debriefings is to learn from previous, collective experience. Learning outcomes may vary among team members and decisions are not necessarily required. Also called after-action reviews, after-event reviews, and post-event reviews, debriefings aim to provide the structure for shifting from automatic/habitual to more conscious/deliberate action and information processing (Ellis and Davidi, 2005; DeRue et al., 2012). Debriefings allow for reflection and self-explanation, data verification and feedback, understanding the relationship between teamwork and task work, uncovering and closing knowledge gaps and disparity in shared cognition, structured information sharing, goal setting and action planning, as well as changes in attitudes, motivation, and self and collective efficacy (Ellis and Davidi, 2005; Rudolph et al., 2007, 2008; DeRue et al., 2012; Eddy et al., 2013; Tannenbaum and Cerasoli, 2013; Tannenbaum and Goldhaber-Fiebert, 2013; Tannenbaum et al., 2013; Kolbe et al., 2015; Eppich et al., 2016; Sawyer et al., 2016b; Allen et al., 2018). In healthcare, debriefings are particularly suited for *ad hoc* teams. While they have become a core ingredient of simulation-based team training (Cheng et al., 2014; Eppich et al., 2015; Sawyer et al., 2016a), their use in daily clinical practice is still limited (Tannenbaum and Goldhaber-Fiebert, 2013) given their vast potential (Mullan et al., 2014; Kessler et al., 2015; Eppich et al., 2016).

The criticality of team process in clinical team debriefings is illustrated in a sample situation in Table 3. This example sheds light on the question how team members and teams as a whole can make use of reflexivity on their team- and taskwork. This includes the issue of identifying process-related markers that indicate turning points in the team process, setting the course for more or less effective team output.

**TABLE 3:** Example of a problematic teamwork situation in clinical debriefings.

**Situation**	**Potential teamwork process problems**	**Required teamwork process insights**
After the management of an unexpected cardiac arrest during surgery, most team members come together for a debriefing. While the participating attending physicians engage in a heated discussion about who was right and who caused the cardiac arrest, the residents and nurses are rather quiet. After a few minutes, the most senior attending physician shares his thoughts on why everybody did what they did and concludes the debriefing, advising the team at large that the mistake simply must not happen again.	Team members may experience fear, anxiety, and embarrassment when making and discussing potential mistakes and engage in face-saving actions such as withdrawal, reluctance to ask for help and disclose errors, and obscuring critique (Schein, 1993; Edmondson, 1999; Rudolph et al., 2013).	Identification of team adaptation mechanisms for creating and maintaining psychologically safe learning moments for clinical debriefings.
	Lack of debriefing rules (Allen et al., 2015; Kolbe et al., 2015), psychological safety and voice (Rudolph et al., 2014).	Understanding of required debriefing rules.
	Risk of shallow or short-sighted argumentation, single rather than double-loop learning, and low levels of reflection and limited effectiveness of feedback (Argyris, 2002; Homayounfar et al., 2015; Kihlgren et al., 2015; Hughes et al., 2016; Boos and Sommer, 2018).	Identification of characteristic modes of argumentation in debriefings depending on status, context, authority gradient and potential turning points and use of structural instabilities in communication.

The three examples were chosen to illustrate generic features of team tasks and team processes. Team tasks call for heterogeneous expertise to be shared, and problem-solving and decision-making procedures that fit task requirements. The tasks require teams to effectively handle interdependent subtasks. And, teams can learn best when they reflect on their team- and taskwork. The vehicle for the accomplishment of all of these task requirements is the team process. The identification of functional team behaviors, critical points and phases in the team process, patterns of how team behavior evolves and adapts to task requirements as well as the facilitation of appropriate team process patterns can help to improve teamwork.

## Previous Methodological Approaches and Their Consequences

After having outlined tasks, prototypical process patterns, and respective research needs in the three team examples, we now put the methodological approaches of how teamwork in these representative examples has mostly been studied centerstage and highlight its consequences. In order to be as specific, illustrative and substantial as possible, we will—in a subsequent step-start from the examples to conceptualize and describe methods that promise deeper and more differentiated insights into teamwork and thus provide a basis for more effective practical interventions. We show important implications of focusing on team dynamics and using suitable methods to capture dynamic processes for team performance outcomes.

### Previous Methodological Approaches of Studying Teamwork in Multidisciplinary Decision-Making Boards

Studies investigating the effectiveness of multidisciplinary decision-making boards have mainly relied on surveys or database review. Database reviews include the systematic review of certain documents, for example hospitals’ patient documentation system. Surveys include questionnaires on specific aspects of self-reported teamwork quality and processes, typically provided by team members in a cross-sectional way. Rating scales such as behavior-anchored rating scales include behavior examples for desired and undesired behavior and a scale for assessing the quality of these behaviors, mostly provided to non-team members (e.g., observers) in a cross-sectional way. Studies using these methods have mostly focused on input and output factors such as (a) whether a multidisciplinary decision-making board is present or not (Keating et al., 2013), (b) whether tumor boards are attended or not (Kehl et al., 2015), (c) the content that is being discussed (Snyder et al., 2017), (d) whether conducting a tumor board leads to a change in management plan or not (Tafe et al., 2015; Brauer et al., 2017; Thenappan et al., 2017), (e) the feasibility with respect to use of technology or overall duration (Marshall et al., 2014), (f) the degree to which the tumor board is valued by participants (Snyder et al., 2017), and (g) the documentation during the board meeting (Farrugia et al., 2015). These studies provide valuable information on the context and some organizational conditions of tumor boards’ effectivity which should not be underestimated (Salas, 2016). However, they are limited in their potential to reveal insights into the actual process and quality of information-sharing and decision-making. This is problematic because it is particularly the quality rather than quantity of communication that is important for performance (Marlow et al., 2018). That is, whereas some effectiveness factors such as optimal team composition, infrastructure, and data base logistics are already well-investigated, there are fewer data on advantageous interaction and communication processes before and during multidisciplinary decision-making board meetings. This is challenging because, as illustrated in the meeting example above, it is particularly the dynamic process that—in interaction with task complexity, time pressure, conflicting goals, and hierarchical structure—endangers the quality of the decision outcome.

Some studies have explicitly addressed the decision-making in tumor boards. They have relied on self-reports (Lamb et al., 2011) and rating tools such as the Multidisciplinary Team Metric for Observation of Decision-Making (MDT-MODe, Lamb et al., 2013; Shah et al., 2014). Although not addressing the decision-making process as such, these studies have provided valuable knowledge on (a) the ability to reach decisions (e.g., 82.2 to 92.7%, Lamb et al., 2013), (b) the attendance rate and duration of case reviews (e.g., 3 min per case, Shah et al., 2014), (c) estimates of the (poor) quality of presented information (e.g., 29.6 to 38.3%, Lamb et al., 2013), (d) estimates of the (poor) quality of teamwork (e.g., 37.8 to 43.0%, Lamb et al., 2013), (e) the comparative quality of team members’ contributions (e.g., highest from surgeons, Shah et al., 2014), and (f) the barriers to reaching decisions (e.g., inadequate information, Lamb et al., 2013). Although the authors of these studies conclude that rating and self-report tools allow for reliably assessing the quality of teamwork and decision-making (e.g., Lamb et al., 2011), we argue that the methodology of these studies does not allow for insights into the actual, dynamic process of information-sharing and decision-making and the quality of the communication process: it remains unanswered (a) how contributions are shared among board members of different levels of hierarchy, (b) who actually contributes when with which information, (c) how other board members react, (d) how individual contributions (not) influence the decision recommendation, and (e) how dissent about evaluations and recommendations emerges and dissolves. We have argued that neglecting these critical characteristics of the decision-making process is to some degree comparable to a patient undergoing surgery while his or her condition is judged using a rating scale from 1 (*bad*) to 5 (*good*) instead of collecting and interpreting data using continuous, machine-based monitoring of heartbeat, breathing, blood pressure, body temperature, and other body functions (Kolbe and Boos, 2018).

### Previous Methodological Approaches of Studying Teamwork in Fast-Paced, Acute Care Settings

A number of studies have been conducted to assess how healthcare teams manage fast-paced, acute care settings. They relied on various methods ranging from surveys (Valentine et al., 2015), over rating tools (e.g., Undre et al., 2009; Couto et al., 2015) to event and time-based observation tools (e.g., Riethmüller et al., 2012; Schmutz et al., 2015; Su et al., 2017). Teamwork observation measures have been developed for capturing teamwork in complex medical situations (e.g., Fletcher et al., 2004; Yule et al., 2006; Manser et al., 2008; Kolbe et al., 2009, 2013a; Tschan et al., 2011b; Kemper et al., 2013; Robertson et al., 2014; Seelandt et al., 2014). Overall, these observation tools fall into two main categories: *behavioral marker systems* (e.g., Fletcher et al., 2004; Yule et al., 2006; Undre et al., 2009; Kemper et al., 2013; Jones et al., 2014; Robertson et al., 2014) and *coding schemes* (e.g., Manser et al., 2008; Kolbe et al., 2009, 2013a; Tschan et al., 2011b; Seelandt et al., 2014). Both types of tools include a number of advantages and disadvantages (Kolbe and Boos, 2018). For examp le, Undre and colleagues applied a behavioral marker system at three designated times during 50 surgical procedures. They found that teamwork behavior could actually be compared between members of different operating room subteams (Undre et al., 2007). They were also able to show that surgeons’ teamwork scores deteriorated toward the end of procedures (Undre et al., 2007). Whereas these results provide valuable knowledge of teamwork estimates and perceived quality, they do not provide insights into the actual operating room team interaction process. This has been possible with studies using behavior coding. For example, Tschan and colleagues continuously coded communication of 167 surgical procedures and found that especially case-irrelevant communication during the closing phase of the procedure was associated with higher rates of surgical site infections (Tschan et al., 2015). Similarly, Riethmüller and colleagues applied a category system for team coordination in anesthesia (Kolbe et al., 2009) for coding coordination activities of simulated anesthesia task episodes and, in addition, assessed awareness for situational triggers and subsequent handling of complications within post-simulation interviews based on stimulated video-recall of the critical phases around the complication. They showed that the occurrence of a complication, e.g., an anaphylaxis or a malign hyperthermia, during a simulated routine anesthesia requires a shift from implicit to explicit coordination behavior (Riethmüller et al., 2012). Also, Weiss and colleagues tested the effects of inclusive leader language on voice in multi-professional healthcare teams in simulated medical emergencies. Specifically, they coded implicit (i.e., First-Person Plural pronouns) and explicit (i.e., invitations and appreciations) inclusive leader language and found that leaders’ implicit leader utterances were more strongly related to residents’ (in- group) and explicit invitations related more strongly to nurses’ (out-group) voice behavior (Weiss et al., 2017a).

As these studies using behavior coding as stand-alone method for capturing teamwork indicate, they—although requiring much time and many resources—do not only provide very specific insights into the relationship between team dynamics and outcomes but also offer actionable knowledge for more targeted team training intervention.

### Previous Methodological Approaches of Studying Teamwork in Clinical Debriefing

The empirical investigation of debriefing and reflexivity in teams is relatively new. Although their overall team context bears similarities with multidisciplinary decision-making boards, research on debriefings has been significantly different from research on the decision-making boards. In disciplines such as psychology and organizational behavior, this research involves experiments (e.g., Gurtner et al., 2007; Ellis et al., 2009, 2010; DeRue et al., 2012; Eddy et al., 2013; Konradt et al., 2015; Otte et al., 2018) and field studies (Vashdi et al., 2013; Weiss et al., 2017b) in which the impact of reflexivity interventions on defined outcomes is tested and different debriefing approaches are compared (e.g., unstructured vs. structured). In disciplines such as healthcare and medical education, there is far more conceptual than empirical work on debriefings. The conceptual work has focused on how to conduct debriefings (Rudolph et al., 2007, 2008, 2013, 2014; Cheng et al., 2014; Eppich et al., 2015, 2016; Kessler et al., 2015; Sawyer et al., 2016a; Cheng et al., 2017; Kolbe and Rudolph, 2018; Endacott et al., 2019). The empirical work has focused on communication in debriefings, albeit rather unsystematically and rarely applying rigorous team science methodology (e.g., Husebø et al., 2013; Kihlgren et al., 2015). Consequences of previous research on teamwork in debriefings include valuable knowledge on debriefing effectiveness and on macro-level debriefing process on the one hand and very limited actionable knowledge on optimal debriefing interaction processes and facilitation for high quality reflection on the other hand.

There are measures available for assessing team reflection and debriefing: (a) REMINT—a reflection measure for individuals and teams (Otte et al., 2017), (b) Debriefing Assessment for Simulation in Healthcare (DASH, The Center for Medical Simulation, 2010; Brett-Fleegler et al., 2012), (c) Objective Structured Assessment of Debriefing (OSAD, Arora et al., 2012), and (d) DECODE for assessing debriefers’ and learners’ communication in debriefings (Seelandt et al., 2018). While REMINT is a self-report measure and not applicable for assessing team dynamics, DASH and OSAD are behavioral marker systems. A recent study pointed to the challenges of measuring team debriefing quality via behavioral markers: Hull and colleagues compared OSAD-based evaluations by examining expert debriefing evaluators, debriefers, and learners (i.e., team members). They found significant differences between these groups: (a) Debriefers perceived the quality of their debriefings more favorably than expert debriefing evaluators. (b) Weak agreement between learner and expert evaluators’ perceptions as well as debriefers’ perceptions were found (Hull et al., 2017). That is, whereas research applying behavioral marker tools can reveal knowledge on differences in perceptions of debriefer/debriefing quality, it provides only limited insights into optimal debriefing interaction processes and how to facilitate high quality reflection in debriefings. This is problematic because, similarly to multidisciplinary decision-making boards (example 1), it is the quality rather than quantity of communication that is important for performance (Marlow et al., 2018); and so far not much is known about how to achieve high quality team interaction during clinical debriefings.

In sum, the review of existing methods used in the three exemplary team research areas shows that approaches for assessing team processes as the critical mechanism mediating the effects of input factors on team performance outcomes exist. Particularly advanced is the research on teamwork in fast-paced, acute care settings with progressive development and application of methods apt for capturing the dynamics of teamwork. Still, overall there is too much focus on aggregate measures, rating tools, and self-report data instead of fine-grained process analysis (Table 4). In what follows, we illustrate potential additional methodological approaches which are, for the time being, more laborious and highlight their consequences with respect to benefits for applied team science. We show the benefits of team interaction process analysis for shedding light on dynamics of teamwork during decision-making in multidisciplinary boards, fast-paced, acute care settings, and during shared reflection.

**TABLE 4:** Previous and laborious methodological approaches and their consequences.

**Example**		**Team conceptual foundation**	**General research question**	**Previous methodological approaches and their consequences**	**Laborious methodological approaches and their consequences**
1	Multi-disciplinary decision-making boards	Collective information sharing and decision-making in *ad hoc*, diverse teams (Stasser and Titus, 1987; Larson et al., 2002; Mesmer-Magnus and DeChurch, 2009; Schulz-Hardt and Mojzisch, 2012).	What are the resulting risks of how input characteristics that are typical of multidisciplinary decision-making boards (e.g., high salience of status and hierarchy, conflicting goals, time pressure) may be associated with ineffective decision-making dynamics and suboptimal results? What are effective countermeasures for managing these risks given the special characteristics of these boards?	*Approaches:* Database reviews Surveys/self-reports Rating scales *Consequences:* + Knowledge about input-output relations + Knowledge about context factors and selected organizational conditions + Knowledge on selected effectiveness criteria such as team composition, infrastructure, data base logistics − Very limited insights into risks of how typical characteristics of multidisciplinary decision-making boards are associated with ineffective information-sharing and decision-making dynamics, management of dissent and suboptimal results − Very limited actionable knowledge for designing effective countermeasures for managing decision-making risks due to special characteristics of these boards.	*Approach:* Event- or time-based coding of each members’ verbal and non-verbal contributions and analysis of board interaction patterns with respect to characteristic input factors and decision outcomes *Consequences:* − Exhaustive behavior coding and data analysis require significantly more time and resources than using surveys/rating scales + Detailed insights into risks of how typical characteristics of multidisciplinary decision-making boards are associated with ineffective information-sharing and decision-making dynamics, management of dissent and suboptimal results + Actionable knowledge for designing effective countermeasures for managing decision-making risks due to special characteristics of these boards
2	Teamwork in fast-paced, acute care settings	Leadership, coordination, and communication in *ad hoc* teams (Gaba et al., 2001; Künzle et al., 2010; Boos et al., 2011; Tschan et al., 2014; Fernandez Castelao et al., 2015; Su et al., 2017).	How does incivility unfold during fast-paced, acute care settings and what are potential team adaptation triggers of civility? What are team adaptation mechanisms for maintaining and regaining functionality despite low civility? What are the enabling social dynamics of voice behavior during fast-paced, acute care settings? How can voice behavior emerge and be effective?	*Approaches:* Surveys/self-reports Rating scales/behavior-marker systems *Consequences*: + Knowledge of perceived teamwork estimates and perceived teamwork quality + Differences in the perceptions of teamwork among team members or subteams highlighted − Very limited actionable knowledge on actual team interaction such as unfolding of incivility and how it relates to performance outcomes *Alternative approach:* Time- and event-based behavior coding *Consequences of alternative approach* − Coding requires significantly more time than using surveys/rating scales + In-depth, actionable knowledge on the process of team interaction and adaptation	*Approach:* Time- and event-based behavior coding combined with social-sensor-based measurement (e.g., physiological data, pose) *Consequences:* − Coding still requires significantly more time than surveys/rating scales +/− Sensor-based measurement is more feasible and unobtrusive but strategies for data analysis are still being developed + Comprehensive, in-depth, actionable knowledge on the dynamic process of actual visible and invisible team interactions related to phenomena such as (in-)civility and voice during fast-paced, acute care settings
3	Post-event, clinical team debriefing	Individual and team learning in *ad hoc* teams (Gurtner et al., 2007; Edmondson, 2012; Tannenbaum et al., 2012; Vashdi et al., 2013; Konradt et al., 2015; Schmutz and Eppich, 2017). Reflective practice (i.e., the exploration of one’s mental routines, taken-for-granted assumptions, and their behavioral consequences) and shared reflection (i.e., collectively looking back on past experience) (Schön, 1983; Argyris, 2002; Edmondson, 2012; Konradt et al., 2016; Koeslag-Kreunen et al., 2018; Otte et al., 2018).	What are team adaptation mechanisms for creating and maintaining psychologically safe learning moments for clinical team debriefings? What are the team interaction processes that constitute high quality reflection? How do structural instabilities in communication (due to status, context, authority gradient) unfold and what are potential turning points in shared reflection? What are the resulting required process rules for conducting clinical debriefings?	*Approach:* Experiments and field studies testing the impact of debriefing or its structure on team outcomes *Consequences:* + Knowledge on debriefing effectiveness and on macro-level debriefing process − Very limited knowledge on optimal debriefing interaction processes − Very limited actionable knowledge on mechanisms for establishing psychological safety in debriefings − Very limited knowledge on how to facilitate high quality reflection *Alternative approach:* Self-reports Rating scales/Behavioral marker systems *Consequences of alternative approach:* +/− Knowledge on differences in perceptions of debriefer/debriefing quality − Very limited knowledge on optimal debriefing interaction processes − Very limited actionable knowledge on mechanisms for establishing psychological safety in debriefings − Very limited knowledge on how to facilitate high quality reflection	*Approach:* Time- and event-based behavior and communication content coding combined with social-sensor-based measurement (e.g., eye-tracking, pose) *Consequences:* − Behavior and communication coding still requires significantly more time than using surveys/rating scales +/− Sensor-based measurement is more feasible and unobtrusive but strategies for data analysis are still being developed + Charting of the information flow by coding utterances, e.g., mention, repeat, value an information + Actionable knowledge on optimal debriefing interaction processes and on mechanisms for establishing psychological safety in debriefings +Actionable knowledge on how to facilitate high quality reflection which can be translated into interventions and process rules for facilitating clinical debriefings

## Laborious Methodological Approaches and Their Benefits

We have labeled the methods we will describe in the following as laborious because they involve, for the time being, more time and resources than most of the above-mentioned approaches. In order to be as specific, illustrative and substantial as possible, we will use the three examples multidisciplinary decision-making boards, fast-paced acute care settings and clinical debriefings to conceptualize and describe methods that promise deeper and more differentiated insights into teamwork and thus provide a basis for more effective practical interventions.

### Laborious Methodological Approaches of Studying Teamwork in Multidisciplinary Decision-Making Boards

In order to complement existing research on multidisciplinary decision-making boards’ effectiveness we recommend to collect data by means of event-based or time-based sampling of critical interaction behavior and to analyze data by applying coding systems which have been designed to help uncovering team decision processes which are critical but invisible for the unaided eye (Table 1). These methods allow for in-depth analysis of what actually happens in multidisciplinary decision-making boards. This is important for identifying success factors. For example, using the Advanced Interaction Analysis for Teams (act4teams) coding scheme (Kauffeld et al., 2018) for analyzing multidisciplinary decision-making board team member behaviors could provide useful insights into (a) the optimal sequence of voicing information versus expressing decision preferences too early in the meeting (Mojzisch and Schulz-Hardt, 2010), (b) the impact of board leaders’ statements compared to lower status members’ contributions on the discussion and outcome (Lehmann-Willenbrock et al., 2015), (c) the emergence and impact of counterproductive meeting behaviors such as arriving late, complaining, and engaging in irrelevant discussions (Allen et al., 2015), and (d) the role of solution-focused meeting behavior such as suggesting a new idea or endorsing a solution (Lehmann-Willenbrock et al., 2017).

Likewise, applying aspects of the Hidden Profile coding scheme (Thürmer et al., 2018), MICRO-CO (Kolbe et al., 2011), or ARGUMENT (Boos and Sommer, 2018) would allow for (a) tracing information processing during the meeting, (b) reveal insights into what and how expert information is actually (not) processed and (not) integrated into decisions, and (c) disassemble the argumentation process into its elements, e.g., identifying grounds that are used to support specific claims for action. In a similar vein, continuously coding actual participation rather than attendance in the meeting would allow for insights into the balance of speaker switches, which has been found to be a predictor of good team performance (Woolley et al., 2010; Lehmann-Willenbrock et al., 2017). These insights into the complex, multi-layered decision-making process will not only be relevant for improving multidisciplinary decision-making boards in healthcare but for multi-team and board decision-making in general.

### Laborious Methodological Approaches of Studying Teamwork in Fast-Paced, Acute Care Settings

To complement existing research and to provide context-sensitive tools for fast-paced, acute care setting, we need methods that capture the very process of teamwork as detailed, sensitive, and unobtrusively as possible. We need actionable knowledge on which behavioral sequences and interaction patterns are effective and which are prone for failure (Lei et al., 2016; Su et al., 2017). As previous research has shown, most of these insights can only be gained with behavior coding and – as new approach in measuring team dynamics – social sensor technology (Rosen et al., 2015, 2018b; Kolbe and Boos, 2018). Behavior coding as stand-alone method for capturing teamwork requires much time and many resources. At the same time, it not only provides very specific insights into the relationship between team dynamics and outcomes that would otherwise remain hidden but also offers actionable knowledge for more targeted team training intervention. As an attempt to more efficiently collect behavioral team data, social sensors have been recently introduced (Dietz et al., 2014; Kozlowski, 2015; Rosen et al., 2015, 2018b; Schmid Mast et al., 2015; Chaffin et al., 2017; Kozlowski and Chao, 2018). They use sensor technology which is, for example, included in smartphones or new types of wearable devices (e.g., smartwatches and bracelets) to measure behavioral cues and process these data to extract behavioral markers of relevant social constructs (Pentland, 2008). On the individual level, potential markers include participants’ body activity, speech consistency, cardiovascular features, or electrodermal activity. On the team level, markers include face-to-face interaction, centrality of certain team members allowing for a social network analysis, interpersonal distance, and behavioral mimicry. As such, social sensors have the potential to provide high-frequency, automated, low-cost, and unobtrusive measurement of behavioral team data (Kozlowski, 2015; Rosen et al., 2015; Chaffin et al., 2017).

The ability to continuously monitor team members might allow for an in-depth analysis of team dynamics, especially during the management of fast-paced, acute care tasks where other forms of data access are limited and potentially intrusive. Respective research in healthcare has revealed promising results. For example, Petrosoniak and colleagues applied an overlay tracing tool to track selected healthcare team members’ movement during 12 high-fidelity *in situ* simulation trauma sessions. They found differences in workflow, movement and space used between team members which provide a deeper understanding of teamwork during managing a medical emergency (Petrosoniak et al., 2018). In another study, Vankipuram and colleagues used radio identification tags and observations to record motion and location of clinical teams and were able to model behavior in critical care environments. That is, the detected behavior could be replayed in virtual reality and provides options for further analysis and training (Vankipuram et al., 2011). More recently, Rosen and colleagues used wearable as well as environmental sensors to capture nurses’ work process data in a surgical intensive care unit and found that the respective measures were able to predict perceived mental and physical exertion and, thus, contribute to the measurement of workload (Rosen et al., 2018c).

With respect to future research, social sensors might be able to capture the very process of teamwork. Especially in fast-paced, acute care settings they can complement traditional measurement methods to provide a more comprehensive analysis of team dynamics and actionable knowledge of which behavioral sequences and interaction patterns are effective (Kannampallil et al., 2011). As social sensors are able to provide information about the development and adaptation of team members’ emotional states, their relative proximity, and their activity level, they could, for example, reveal insights into (a) the development of stress levels among team members while (not) speaking up (e.g., changes in heart frequency or electrodermal activity, Setz et al., 2010) and potential countermeasures, (b) the potential of mimicry by team members for revealing civility while speaking up (Chartrand and Bargh, 1999; Meyer et al., 2016), (c) the proximity and centrality of team members as enablers or barriers for speaking up (Jackson and Hogg, 2010), (d) the development of adaptive coordination, especially switching from implicitness to explicitness, as a trainable skill set (Riethmüller et al., 2012).

Again, this kind of results would provide actionable knowledge on the dynamics of leadership and voice which can be used in team trainings. Facing medical emergencies, teams must act immediately, fast and in a highly efficient manner as emergencies often times imply a life-or-death-struggle. Methods are required that can grasp the criticality of situational triggers in the flow of a routine process, the sensitivity and situational awareness thereof and the accurate fitting of well-coordinated behavior for an efficient task management.

### Laborious Methodological Approaches of Studying Teamwork in Clinical Debriefings

To complement existing research on team debriefing processes and effectiveness we recommend to collect data by means of event-based or time-based sampling of interaction behavior and to analyze data by applying coding systems which have been designed to help uncovering conversational team learning processes (Table 3). For example, using DECODE—the coding scheme for assessing debriefers’ and learners’ communication in debriefings (Seelandt et al., 2018) or the act4teams Coding Scheme (Kauffeld et al., 2018) for analyzing debriefing communication behavior could provide useful insights into the debriefings’ ideal macro (e.g., reaction phase, analysis phase, summary phases, Rudolph et al., 2007) as well as micro structure (e.g., what kind of facilitator’s communication behaviors trigger group members’ reflection statements, Husebø et al., 2013), in particular with respect to feedback and inquiry (Rudolph et al., 2007; Hughes et al., 2016; Kolbe et al., 2016). It could inform the potential association of team members’ status, professional discipline, actual profession, and their contributions to the debriefing discussion (Lehmann-Willenbrock et al., 2015), the emergence and impact of counterproductive debriefing behaviors such as arriving late, complaining, lecturing, and engaging in irrelevant discussions (Allen et al., 2015, 2018; Kolbe et al., 2015), the optimal balance of understanding and exploring vs. engaging in finding solutions (Kolbe et al., 2015), characteristic modes of argumentation in debriefings depending on status, context, authority gradient, and potential turning points and use of structural instabilities in communication, and the role of leadership in debriefing discussions (Koeslag-Kreunen et al., 2018). Similarly to proposed multidisciplinary decision-making boards research, capturing actual participation rather than attendance in the debriefing would allow for insights into the balance of speaker switches, which has been found to be a predictor of good team performance (Woolley et al., 2010; Lehmann-Willenbrock et al., 2017).

With respect to future research, behavior coding of team debriefings might be complemented with other data collection technology. For example, using eye tracking technology (Hess et al., 2018) might reveal insights the role of eye-contact for establishing and maintaining psychological safety in debriefings.

## Conclusion

We have contrasted methodological approaches for studying team dynamics and their consequences. Given the increasing use of teams in modern organizations, there is a need to develop and apply scientifically-rooted concepts and methods to grasp team process dynamics as a means to gain a deeper understanding of successful teamwork.

Coding interaction and communication processes in teams based on generic or tailor-made category systems provides benefits for the science of teams. First, a process- and behavior-oriented approach enables us to operationalize theoretical constructs and everyday phenomena such as decision-making, coordination, and reflexivity in a clear-cut manner. Second, focusing on the processual enactment of team phenomena allows for a much richer picture of how they emerge, develop, and interact, how effective patterns evolve, and for identifying breaking points for potential intervention (Wageman et al., 2009). Third, studying team dynamics via behavior observation allows for taking the so-called functional perspective of group research seriously: opening the *black box* of team process as a mediator between input and output factors (Roe, 2011, 2014). For now, team behavior coding is still laborious. New developments in machine learning are likely to significantly reduce the involved workload in the future (Bonito and Keyton, 2018).

Implications of this research will be meaningful for team training and the design of prevention and intervention concepts to improve teamwork. Structural changes of input factors such as team composition, resources, reward systems, and norms can improve teamwork to some degree. But in the end, for determining what makes these changes effective or not, a look into how they are enacted during the team process is necessary. In this manuscript, we have tried to elaborate research questions in the realm of healthcare teams which cannot be answered sufficiently without taking the process of team communication and interaction into consideration. We are convinced that—as in other disciplines—innovation and progress in team research heavily depend on methodological and technological innovation. This is what Gigerenzer (1991) called the “tools-to-theories heuristic.” It is not so much the theories and data that drive scientists to new ideas and the solution of existing problems, but instruments, techniques, and methodical skills (Gigerenzer, 1994). With an increasing innovation grade in team research, we have methods and technology available that allow for much deeper and finer-grained team research and for exploring groundbreaking, new questions.

## Author Contributions

All authors listed have made a substantial, direct and intellectual contribution to the work, and approved it for publication.

## Conflict of Interest Statement

The authors declare that the research was conducted in the absence of any commercial or financial relationships that could be construed as a potential conflict of interest.
